# Supplementary material for: GAVISUNK: genome assembly validation via inter-SUNK distances in Oxford Nanopore reads
Source: Bioinformatics. 2022 Nov 2;39(1):btac714. doi: 10.1093/bioinformatics/btac714 (PMC9805576; doi:10.1093/bioinformatics/btac714)
Supplement: btac714_Supplementary_Data [file btac714_supplementary_data.zip › btac714_Supplementary_Data/GAVISUNK_20220923_Figs_S1-S6.docx]

**
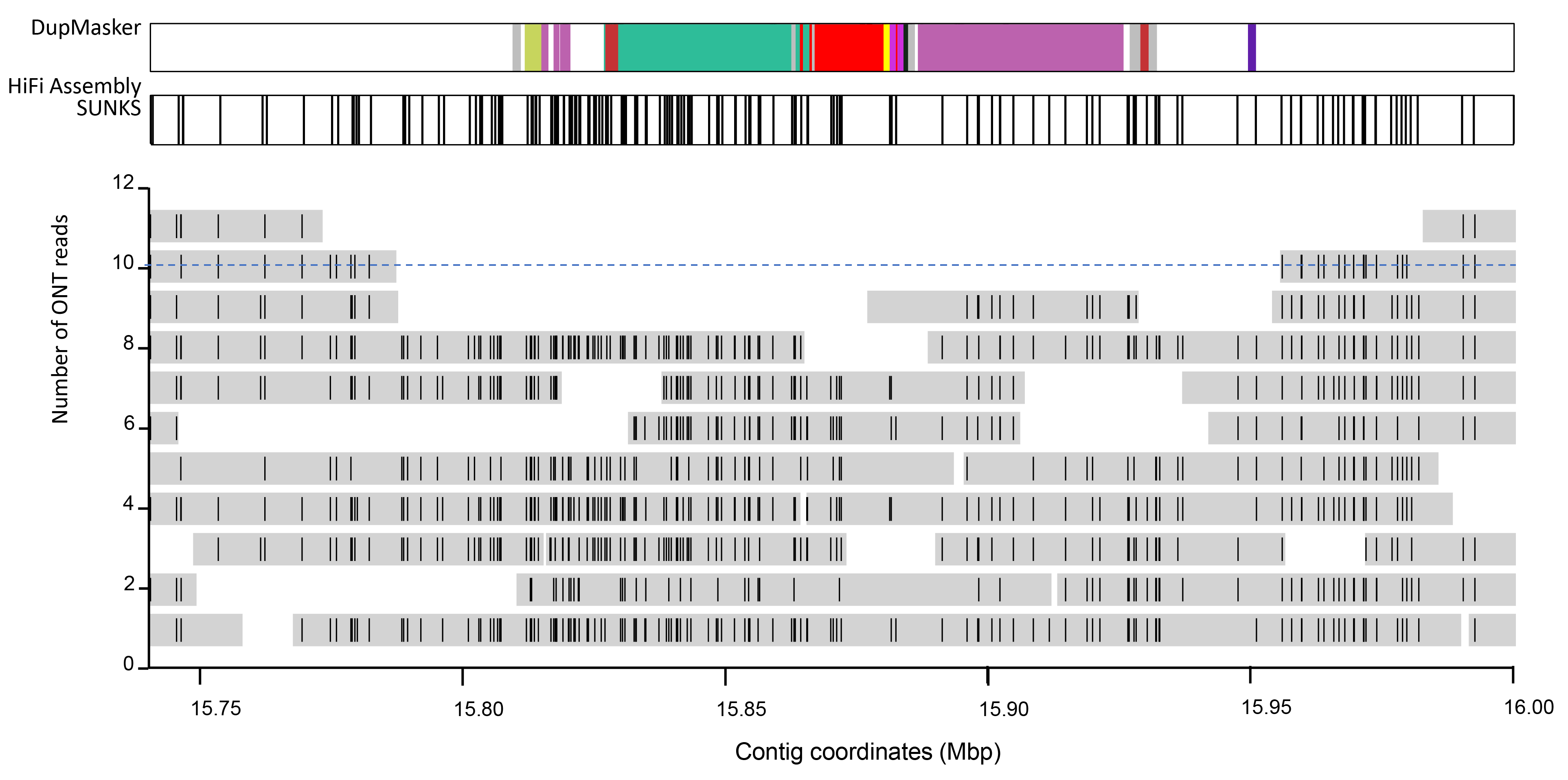
**

**Supplementary Figure 1.**  An example of a validated region within the maternal haplotype assembly of HG00733. Horizontal gray bars represent individual ONT reads, and vertical black lines indicate the position of SUNKs originally detected from the HiFi assembly. All possible assembly SUNKs are shown above, along with a DupMasker track marking segmental duplications. The dotted blue line indicates the mean genome-wide coverage of ONT sequencing data.

`


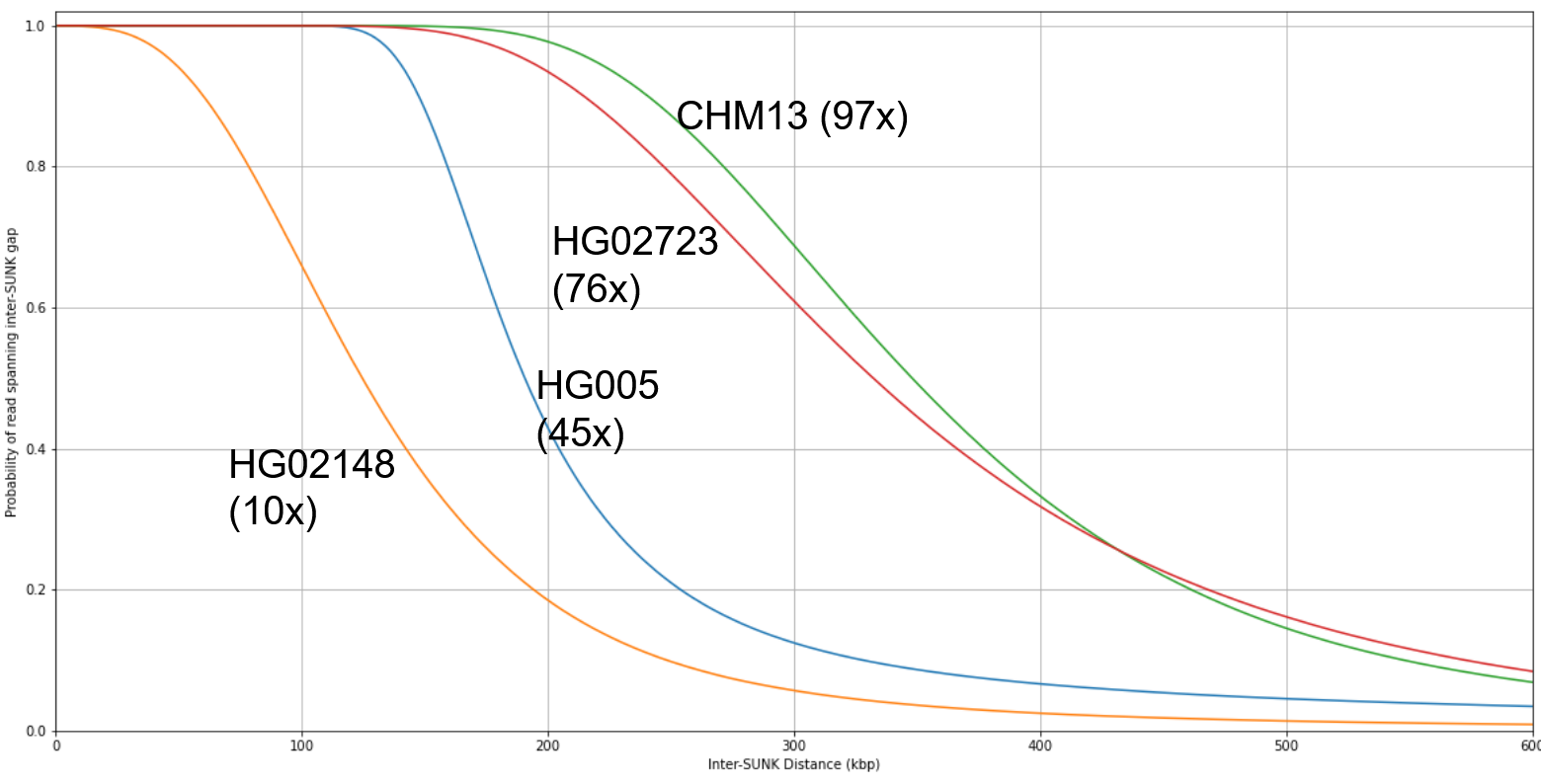


**Supplementary Figure 2. Simulation of** **probability of spanning inter-SUNK gaps.** The probability of reads spanning a given inter-SUNK distance is determined on a per-sample basis by calculating the Poisson distribution of ONT reads across the genome, adjusting for sequencing accuracy and size of the SUNK group. Haplotype-specific ONT sequencing depth is shown in parentheses for each sample.

**
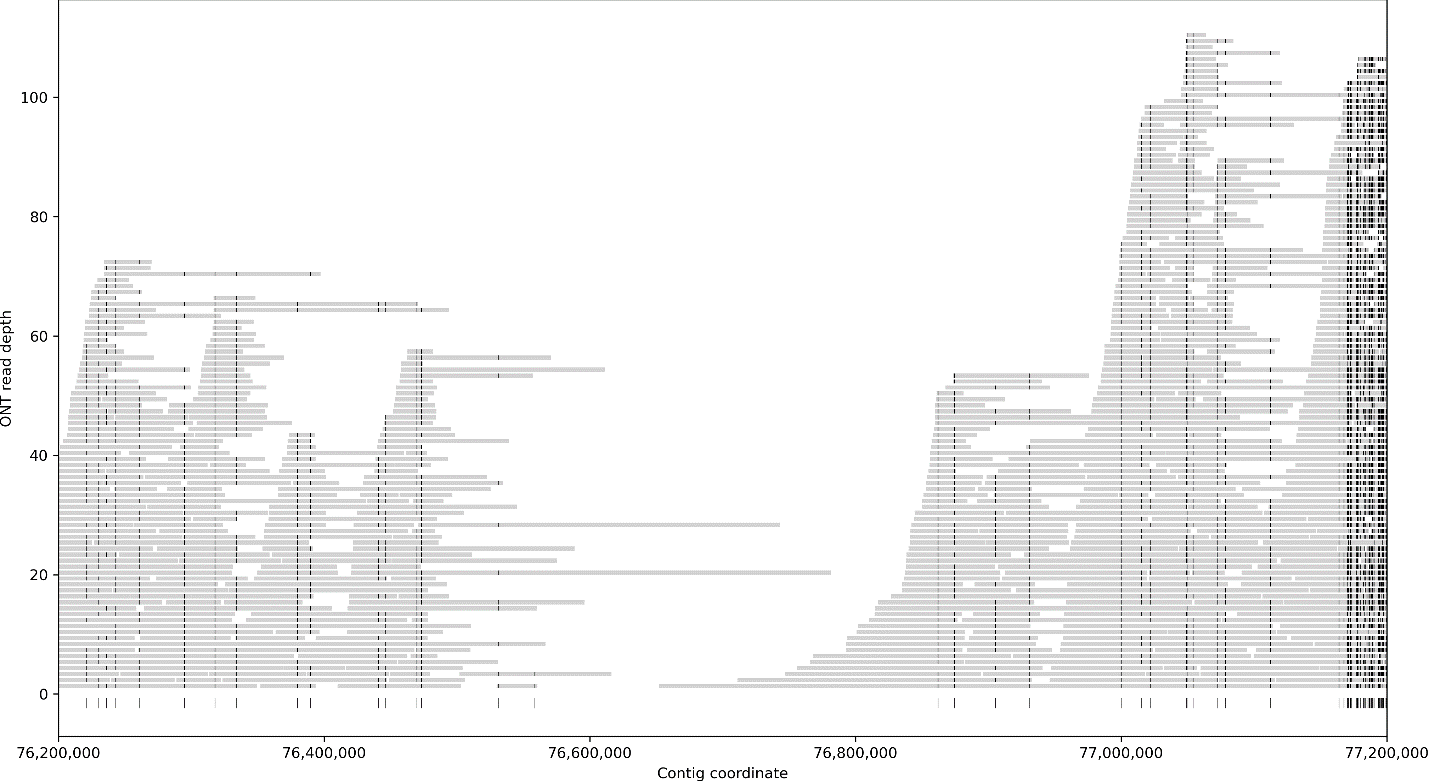
Supplementary Figure 3. Unspannable gap in CHM13-T2T *HYDIN*.** Gray bars with black tickmarks represent ONT reads and their respective SUNKs, while black tickmarks below represent all assembly SUNKs. Because the SUNK density at this recently duplicated locus is too low for ONT reads at the given length and coverage to span between SUNK groups, no determination can be made of assembly correctness.


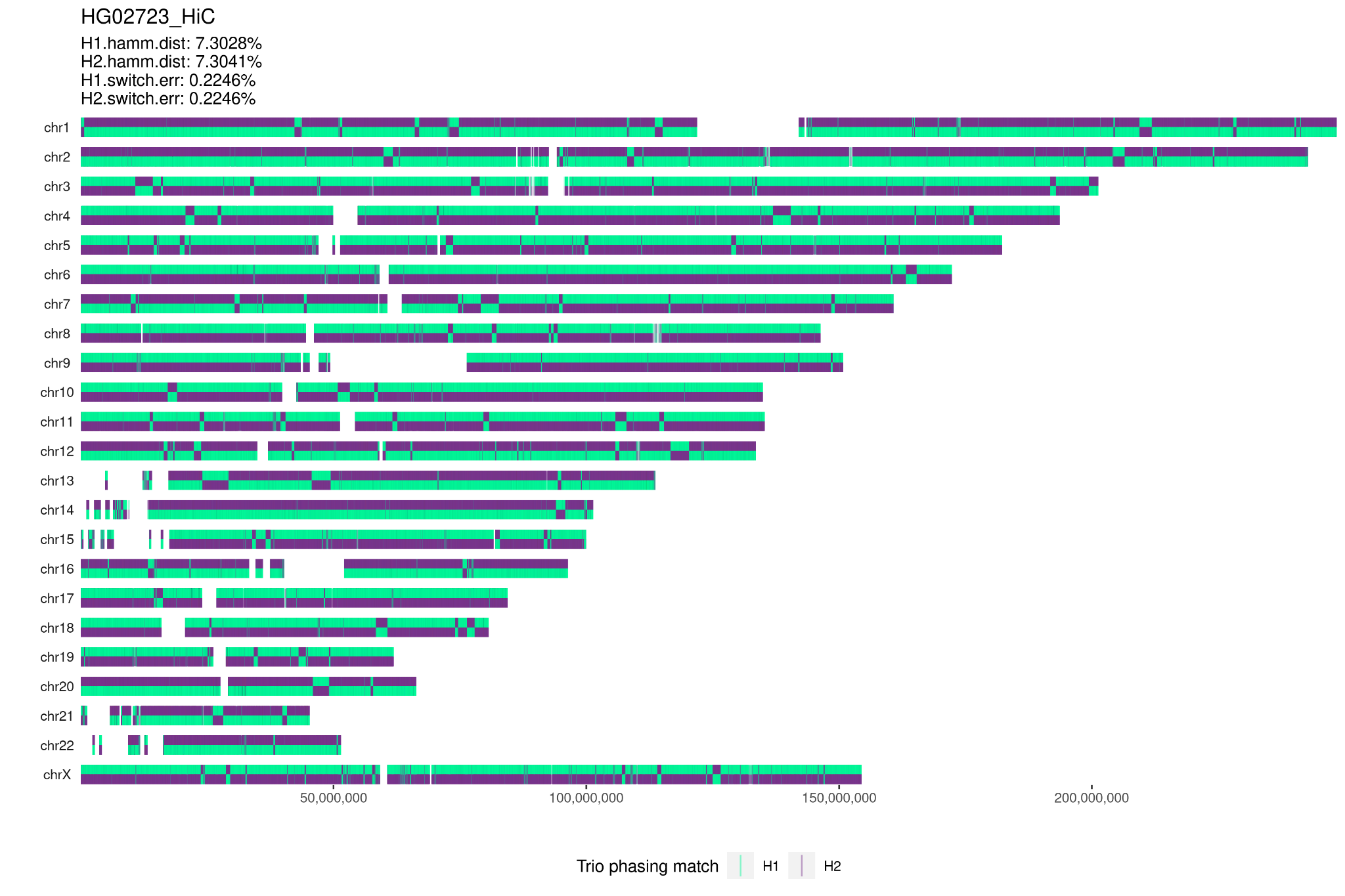


**B**

**A**


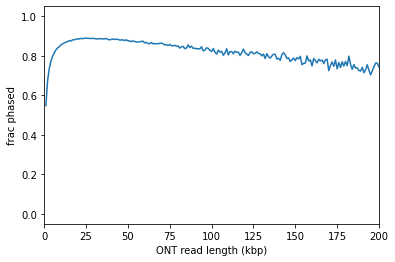


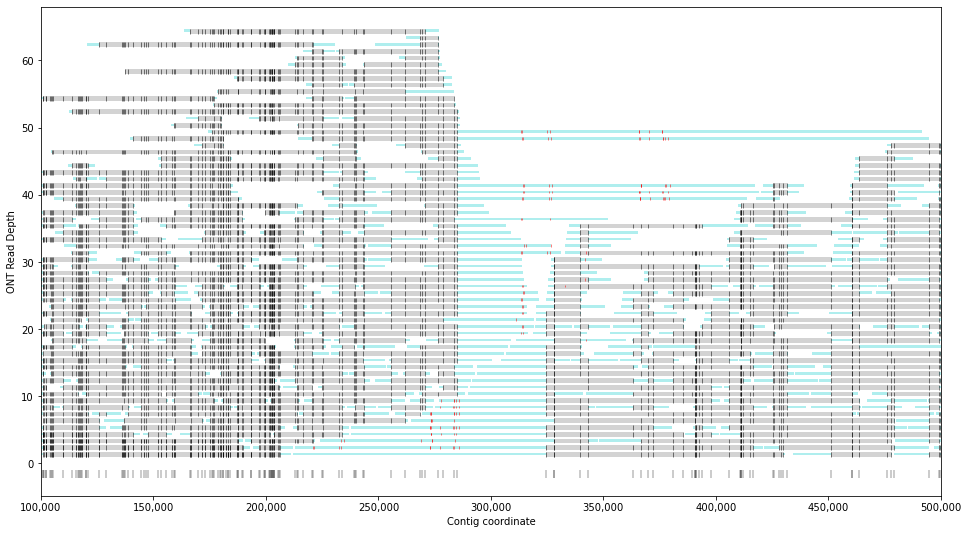


**C**

**Supplementary Figure 4. Human genome sample analysis without parental phasing data.** a) HiC-based phasing of HG02723 evaluated with parental data post hoc b) Phasing of ONT data as a function of ONT read length from the same sample using custom script, HiCphaseONT. Phased ONT data can now be used for GAVISUNK. c) AMY test region phased with Hi-C instead of parental Illumina data.


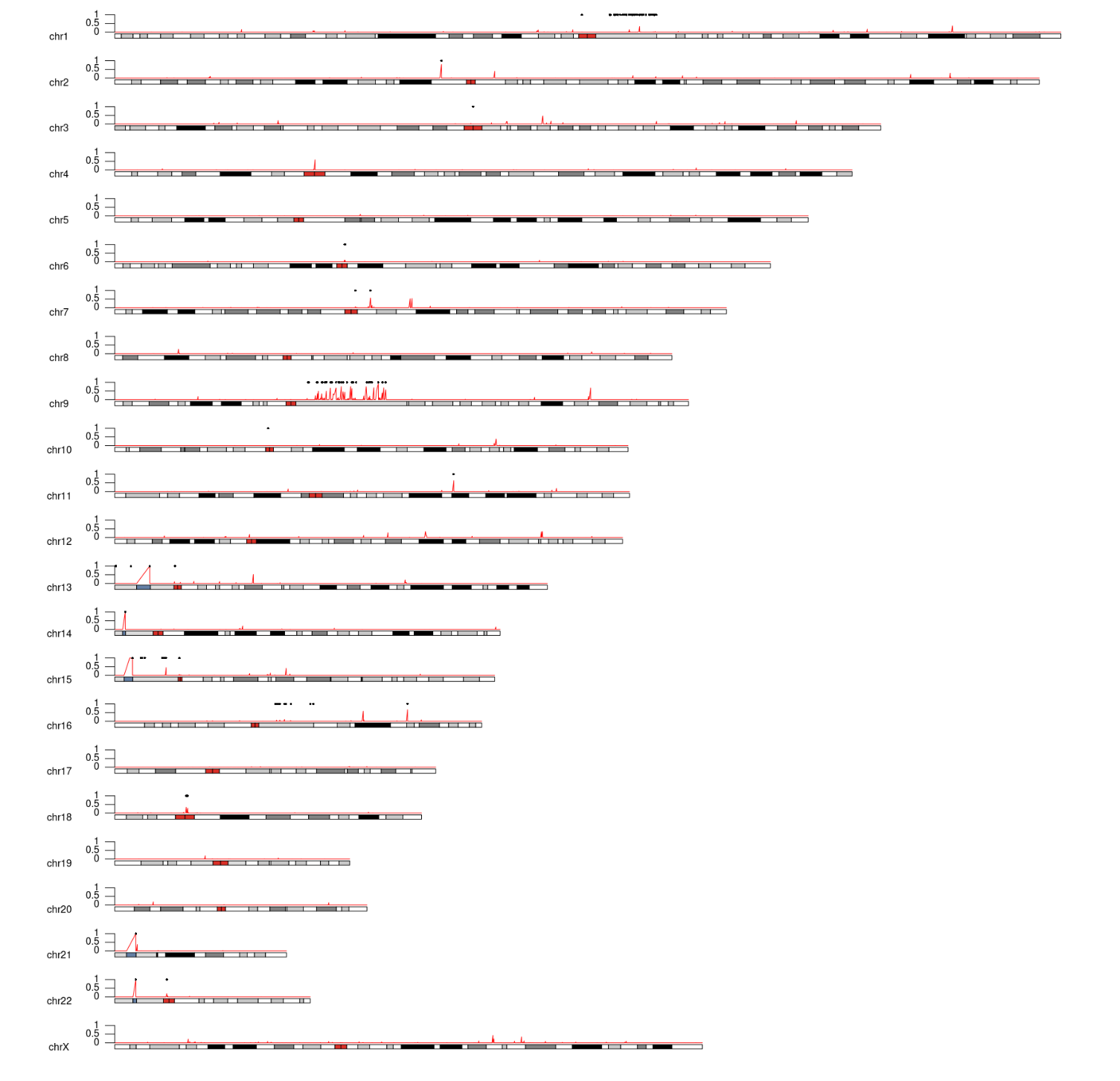


**Supplementary Figure S5: CHM13-T2T empirical validation gaps**. Each dot is an empirical validation gap, while the red line indicates the estimated probability of failing to span an inter-SUNK distance. Validation gaps with high simulated probabilities of coverage fall in the qh regions of chromosomes 1, 9, and 16, rDNA arrays, and centromeres.


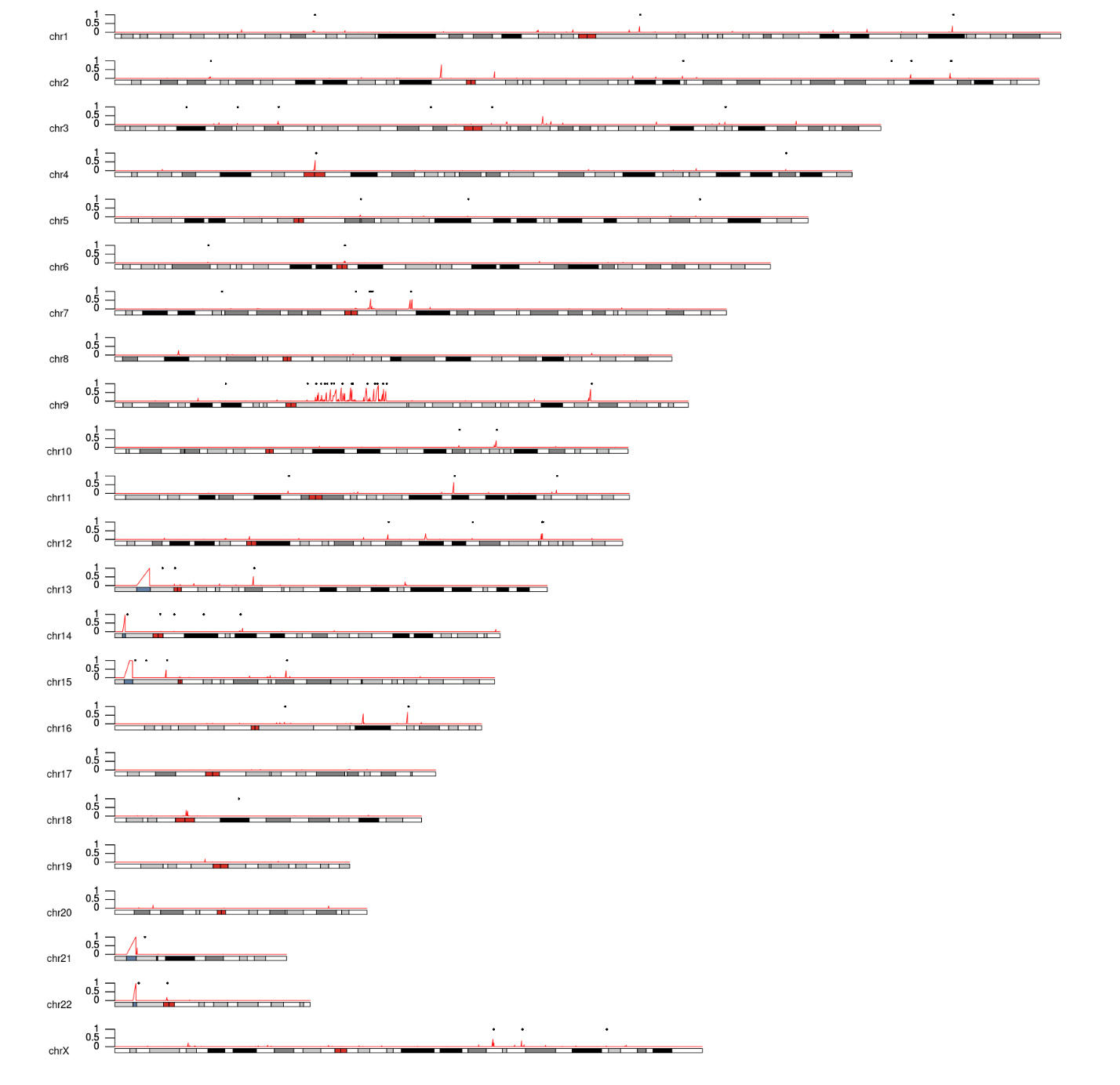


**Supplementary Figure S6: CHM13-T2T simulated validation gaps**. Each dot is a simulated validation gap, while the red line indicates the estimated probability of failing to span an inter-SUNK distance.
